# Association Between Phosphorylated AXL Expression and Survival in Patients with Gastric Cancer

**DOI:** 10.3390/jcm13226694

**Published:** 2024-11-07

**Authors:** Hua Ho, Chiao-Yin Cheng, Chun-Yen Huang, Sheng-En Chu, Yao-Jen Liang, Jen-Tang Sun, Yen-Lin Chen

**Affiliations:** 1 Department of Emergency Medicine, Far Eastern Memorial Hospital, New Taipei 220, Taiwan; h790317@gmail.com (H.H.); meureka@gmail.com (C.-Y.H.); ianchu300@msn.com (S.-E.C.); 2 Graduate Institute of Applied Science and Engineering, Fu-Jen Catholic University, New Taipei 242, Taiwan; chiaoyin810406@gmail.com (C.-Y.C.); 071558@mail.fju.edu.tw (Y.-J.L.); 3 Department of Emergency Medicine, National Taiwan University Hospital Yun-Lin Branch, Douliu City 640, Taiwan; 4 Department of Pathology, Tri-Service General Hospital, National Defense Medical Center, Taipei 114, Taiwan

**Keywords:** phosphorylated AXL, gastric cancer, epithelial–mesenchymal transition, fibronectin, AKT

## Abstract

**Background:** Gastric cancer (GC) is a leading cause of cancer-related mortality, particularly in East Asia. Despite treatment advances, the prognosis remains poor owing to late diagnosis and high metastatic potential. Phosphorylated AXL (pAXL), a receptor tyrosine kinase, promotes cancer progression, including epithelial–mesenchymal transition (EMT), tumor growth, and metastasis. In this study, we aimed to investigate the relationship between pAXL expression and prognosis in patients with GC, focusing on survival outcomes and other biomarkers such as fibronectin and phosphorylated AKT (pAkt). **Methods:** Immunohistochemistry was performed to assess the expression of pAXL, fibronectin, and pAkt in 188 GC specimens collected between 2000 and 2013. H-scores were calculated based on staining intensity and percentage. The association between pAXL expression and patient outcomes was assessed using Kaplan–Meier survival analysis and multivariate logistic regression. **Results:** Higher pAXL expression was significantly associated with improved survival, particularly in male patients. pAXL expression positively correlated with fibronectin and pAkt upregulation, suggesting its role in promoting tumor invasion and EMT. Multivariate analysis identified pAXL, fibronectin, and pAkt as significant prognostic indicators, whereas other factors such as age, tumor grade, and tumor size were not statistically significant. **Conclusions:** This study identified pAXL as a valuable prognostic marker in GC, with higher expression levels associated with better survival outcomes, particularly in male patients. pAXL enhanced the invasive potential of GC cells through fibronectin and pAkt regulation, making it a promising therapeutic target. Further research is needed to explore the potential of pAXL-targeted therapies and better understand their role in cancer progression and treatment response.

## 1. Introduction

Gastric cancer (GC) continues to be a major cause of cancer-related deaths worldwide, despite its declining incidence over the past century. The disease remains most prevalent in East Asia, where the highest rates of prevalence are observed. Globally, approximately 990,000 new cases of GC are diagnosed annually, resulting in approximately 738,000 deaths. Key risk factors for developing GC include older age, male sex, smoking, family history, and genetic predisposition [1,2].

Recent research has focused on identifying molecular pathways that drive GC progression, including genes involved in proliferation, apoptosis, invasion, and metastasis. Among them, caspase-3 is a key enzyme in both apoptosis and pyroptosis and induces cell death by cleaving various substrates. In apoptosis, it drives the disassembly of the cell, whereas in pyroptosis, it cleaves GSDME, leading to cell membrane rupture. In addition to cell death, caspase-3 plays a role in regulating growth, differentiation, and tissue homeostasis in both healthy and malignant cells, thereby affecting the tumor response to therapy. It also participates in tissue regeneration and neural development, making it a potential therapeutic target for cancer, heart failure, and neurodegenerative disorders [3]. Another biomarker, Ki-67, is a widely recognized biomarker for cell proliferation with the potential for use in personalized treatment strategies, particularly in renal and GCs. While its role in breast cancer prognosis is well documented, its relevance in renal cancer remains less explored, even though renal cancer cases are rising globally. Recent studies suggest that Ki-67 expression correlates with tumor stage and metastasis in renal cancer, indicating its potential as a therapeutic target. Despite its importance in cancer development, Ki-67 is not essential for cell proliferation but is crucial for chromatin regulation and cancer cell adaptation. A meta-analysis found that high Ki-67 expression is associated with poorer disease-free and overall survival, underscoring its potential as a predictive biomarker for prognosis and treatment selection. Although therapies targeting Ki-67 are still in preclinical phases, further exploration of its molecular mechanisms may enhance cancer management strategies [4,5,6].

ERK (extracellular signal-regulated kinase) is a key component of the Ras/Raf/MEK/ERK (MAPK) signaling cascade, regulating essential cellular functions such as proliferation, survival, growth, metabolism, migration, and differentiation. Activated ERK phosphorylates various substrates and modulates gene expression and protein synthesis, maintaining cellular homeostasis. Dysregulation of ERK signaling plays a critical role in cancer development by promoting uncontrolled cell proliferation and survival, often due to mutations or overactivation of upstream components, such as Ras or Raf. Therefore, ERK is considered a crucial target for cancer therapies, with inhibitors targeting the Ras/Raf/MEK/ERK pathway being investigated for their potential to block tumor growth. Despite progress in understanding the functions of ERK, its complex regulatory mechanisms remain a focus of ongoing research [7,8,9].

STAT3 (signal transducer and activator of transcription 3) is a key transcription factor involved in cancer progression and metabolism. Upon activation by cytokines, growth factors, and other signals, STAT3 dimerizes and translocates to the nucleus where it regulates the expression of numerous genes involved in cell proliferation, survival, migration, and invasion. In cancer, STAT3 is frequently hyperactive due to upstream mutations or elevated cytokine levels in the tumor microenvironment, driving oncogenesis and metastasis. Additionally, STAT3 is involved in reprogramming cancer cell metabolism, affecting pathways such as aerobic glycolysis, oxidative phosphorylation, and mitochondrial function. It influences not only cancer cells but also immune cells and adipocytes within the tumor microenvironment. Given its role in regulating both normal and cancer stem cell functions, STAT3 is a promising target for cancer therapies. However, despite advances in understanding its mechanisms, clinically relevant STAT3 inhibitors are still in development, with ongoing efforts to harness their therapeutic potential [10,11,12,13].

Several antigens, such as Kiel 67 (Ki-67), pAkt, phosphorylated extracellular signal-regulated kinases (pErk), and phosphorylated signal transducer and activator of transcription 3 (pStat3), are involved in promoting cell proliferation and survival, with high expression commonly observed in aggressive cancers [14,15,16]. CD31 and fibronectin are crucial for angiogenesis and metastasis, facilitating tumor growth and spread [17]. E-cadherin and N-cadherin are key players in cell adhesion and epithelial–mesenchymal transition (EMT), and their differential expression indicates an increased metastatic potential [18,19]. Phosphorylated 5‘ AMP-activated protein kinase (pAMPK) and DNAJB4 play roles in cellular stress responses, where pAMPK can have both tumor-suppressive and tumor-promoting effects [20,21]. HMGA1 and MCRS1 are involved in chromatin remodeling and are associated with tumor aggressiveness and poor prognosis. Similarly, HOXA5 and IGF2BP1 regulate gene expression, with IGF2BP1 enhancing tumor growth and metastasis by stabilizing mRNA [22,23]. Additionally, EphA5 influences tumor progression through various signaling pathways, highlighting the intricate network of molecular interactions in cancer development and progression [24].

AXL, a receptor tyrosine kinase belonging to the TAM family, which also includes TYRO3 and MER, regulates various cellular processes such as survival, proliferation, migration, and immune modulation [25,26]. Its activation, primarily triggered by binding with the ligand growth arrest-specific 6 (GAS6), initiates dimerization and autophosphorylation, activating downstream signaling pathways, including PI3K/AKT, MAPK/ERK, and NF-κB [27,28]. These pathways contribute to the survival, motility, and metastasis of cancer cells. Additionally, AXL can be activated through ligand-independent mechanisms, often involving interactions with other receptors such as EGFR and HER2, further promoting oncogenic signaling [25]. AXL overexpression is commonly observed in several types of cancer, including breast, lung, ovarian, and renal cell carcinomas. Its overexpression is often linked to poor prognosis and increased resistance to therapies [25,26,27,28].

Post-translational modifications (PTMs) like phosphorylation regulate cellular processes such as cell growth, differentiation, apoptosis, and cell signaling. The dysregulation of phosphorylation pathways, including those involving AXL, is linked to cancer [9]. The role of AXL in phosphorylating key substrates, like MIG6, shows its influence in pathways critical to tumorigenesis and cancer cell survival [29].

In GC, AXL plays a pivotal role in promoting tumor growth, invasion, and metastasis. Studies have demonstrated that AXL is overexpressed in GC cell lines, with high AXL levels correlating with worse overall survival in patients. The GAS6/AXL signaling pathway facilitates EMT by upregulating the transcription factor ZEB1, enhancing the invasive and proliferative potential of GC cells [30,31].

Previous studies have analyzed AXL expression and survival outcomes in human GC specimens, but they have paid limited attention to patient demographics and clinical characteristics. Therefore, in this study, we aimed to conduct a comprehensive analysis of AXL in relation to patient age, sex, differentiation grade, invasion depth, and relevant gene expression using GC specimens. This study will offer a more complete understanding of survival outcomes, thereby informing potential therapeutic strategies.

## 2. Materials and Methods

### 2.1. Patient Recruitment, Ethics, and Sample Collection

We collected 188 GC specimens from patients between 2000 and 2013 and recorded their age, sex, differentiation grade, tumor size, and staging results. This study was approved by the Institutional Review Board of the Cardinal Tien Hospital (approval number CTH-101-3-5-054).

### 2.2. Tissue Preparation and Array Construction

All the specimens were fixed in formalin and embedded in paraffin. We extracted 2 mm diameter cylindrical specimens and rearranged them into tissue arrays. Finally, the samples were cut into 5 µm thick sections for analysis.

### 2.3. Immunohistochemistry (IHC) Staining Procedure

Each antibody was initially stained using various protocols on a mixed tissue slide, with variations in dilution, incubation time, and antigen retrieval. The dilution conditions were 1:50, 1:100, and 1:200, the incubation times were 16, 32, and 64 min, and antigen retrieval was performed using a heating method with durations of 24, 32, and 48 min. An experienced pathologist reviewed the slides to select the optimal staining conditions based on the staining intensity and non-specific background staining. Once the optimal staining conditions were determined, all subsequent slides were stained using the same protocol.

### 2.4. Antibody Optimization and Staining Protocols

Tissue blocks were sectioned and subjected to immunohistochemistry using the BenchMark XT automated stainer (Ventana, Tucson, AZ, USA). Briefly, consecutive 5 μm thick sections were cut from formalin-fixed paraffin-embedded tissues, mounted on silanized slides, and air-dried at room temperature. An automated stainer was used for deparaffinization, rehydration, and antigen retrieval using Ethylenediaminetetraacetic acid (EDTA) for either 16, 32, or 64 min, depending on the antibody conditions.

The slides were then incubated with primary antibody at 37 °C for 24, 32, or 48 min. After washing three times in buffer, the slides were incubated with a secondary antibody (Universal DAB Detection Kit; Ventana, Tucson, AZ, USA). Tissue staining was visualized using a 3,3′-Diaminobenzidine (DAB) chromogen solution, followed by hematoxylin counterstaining, dehydration, and coverslipping.

The tissue sections were stained with various primary antibodies using an automated immunohistochemistry stainer, with antibody concentrations ranging from 1:50 to 1:500. The antibody details are provided in Supplementary Table S1.

### 2.5. Staining Scoring and H-Score Calculation

**Figure 1:**
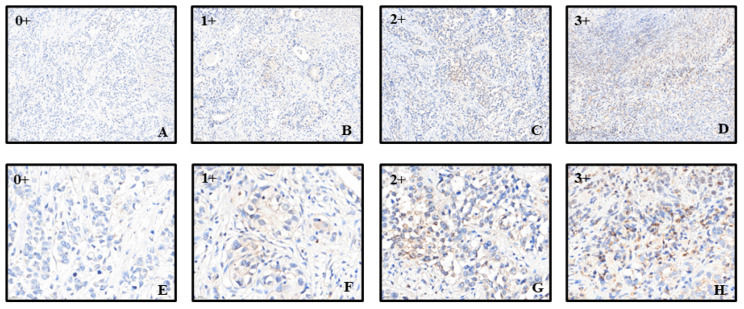
Schematic of phosphorylated AKL (pAXL) immunostaining with different scores. (**A**–**D**) pAXL expression, with the lowest expression scored as 0 and the highest expression scored as 3. The scale bar represents 500 μm; (**E**–**H**) magnified views of the different scores, with the scale bar representing 200 μm.

The staining intensity was scored from 0+ (no staining) to 3+ (strong staining). The biopsy samples represented Stage III GC tissue (Figure 1). The staining percentage for each sample was also recorded. The H-score, which ranges from 0 to 300, was calculated by multiplying the staining intensity by the staining percentage for each sample. For example, an H-score of 300 indicates 3+ staining intensity with 100% staining of the sample. Initially, we attempted to group GC samples based on the median expression of pAXL and ultimately found that an H-score of 3.1 was the optimal cutoff point for pAXL.

### 2.6. Statistical Analysis

To assess the overall fit of the model, we conducted the Hosmer–Lemeshow goodness-of-fit test, which indicated a good model fit with no significant deviation. Statistical analyses were conducted using IBM SPSS 26.0. The Kolmogorov–Smirnov test was applied to assess the normality of the distributions, and since all continuous variables were non-normally distributed, we expressed the H-scores using the median and interquartile range and analyzed them using the U-test. Categorical variables were analyzed using the chi-square test. Variance inflation factor (VIF) was used to examine multicollinearity. If any variable had a VIF value exceeding 10, it would indicate high collinearity between the variables. However, the VIF values for the predictor variables included in our model ranged from 1.03 and 2.43, suggesting that multicollinearity was not an issue. Univariate logistic regression analysis was performed for each variable, and variables showing significant differences were included in a multivariate logistic regression analysis, with age and sex forcibly included. Kaplan–Meier survival analysis was used to evaluate survival outcomes based on high and low expression levels of pAXL. Statistical significance was set at *p* < 0.05

## 3. Results

Table 1 presents a comparison of patient characteristics and tumor features between the low- and high-pAXL-expression groups of patients with GC. A trend toward a larger proportion of female patients was observed in the high-pAXL-expression group (41.3%) than in the low-pAXL-expression group (28.0%); however, this difference was not significant (*p* = 0.067). The median age was non-significantly (*p* = 0.144) lower in the high-expression group (72.5 years) than that in the low-expression group (76 years). The distribution of tumor grades was similar between the two groups (*p* = 0.835), with the majority of tumors being poorly differentiated (72.4% in the low- and 69.4% in the high-expression groups). The median tumor size was comparable between the low (4.5 cm)- and high (4.3 cm)-expression groups (*p* = 0.449). Additionally, although a larger proportion of patients with high pAXL expression were in the early stages (I and II) of GC (47.6%) than those with low pAXL expression (38.4%), the difference was not significant (*p* = 0.226). Overall, although there were observable trends in the distribution of sex, age, and tumor grade, size, and stage between the low- and high-pAXL-expression groups, none of these differences was significant in this cohort of patients with GC (Table 1).

Next, we explored the effects of pAXL expression on other proteins. Table 2 presents a comparison of biomarker expression levels between the low- and high-pAXL-expression groups among patients with GC and highlights significant differences in the expression levels of several markers. In addition, Supplementary Figure S1 illustrates various markers in the context of high and low pAXL expression. Caspase3 and Ki-67 showed similar expression levels between groups, with no significant differences. CD31 and E-cadherin expression levels were slightly higher in the high-pAXL-expression group, although this difference was not significant. N-cadherin expression levels were also similar between the groups. Furthermore, fibronectin, pAkt, pErk, pStat3, and HMGA1 expression levels were significantly higher in the high-pAXL-expression group, with *p*-values of <0.001, <0.001, 0.002, 0.021, and <0.001, respectively. In contrast, the pAMPK and IGF2BP1 levels were significantly higher in the low-pAXL-expression group (*p* = 0.030 and *p* = 0.014, respectively). DNAJB4, HOXA5, MCRS1, and EhpA5DrTT showed no significant differences in expression between groups. These findings revealed that several biomarkers, particularly fibronectin, pAkt, pErk, pStat3, pAMPK, HMGA1, and IGF2BP1, exhibited significant differences in expression based on the pAXL status in patients with GC.

**Figure 2:**
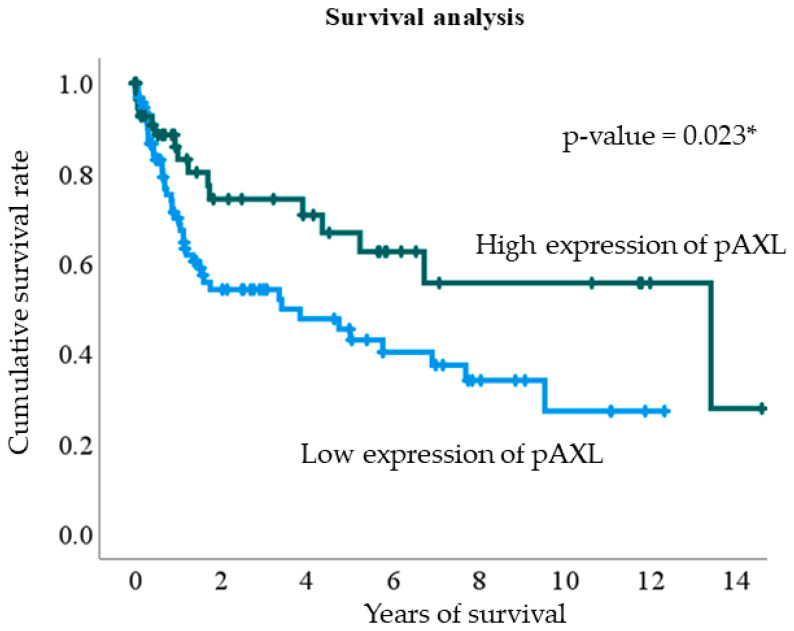
Kaplan–Meier survival analysis plot of phosphorylated AXL (pAXL) expression levels. Blue and green lines represent low and high pAXL expression, respectively. * *p* < 0.05.

The Kaplan–Meier survival curve illustrates the cumulative survival of patients with GC with high pAXL expression (dark green curve) compared to those with low expression (light blue curve). The patients with low pAXL expression had a lower cumulative survival rate over time with a more rapid decline, indicating a higher mortality rate. In contrast, patients with high pAXL expression exhibited a better survival rate and a slower decline in cumulative survival. This analysis suggests that high pAXL expression is associated with improved survival outcomes compared to low pAXL expression in patients with GC. The log-rank test *p*-value was 0.023 (Figure 2).

**Figure 3:**
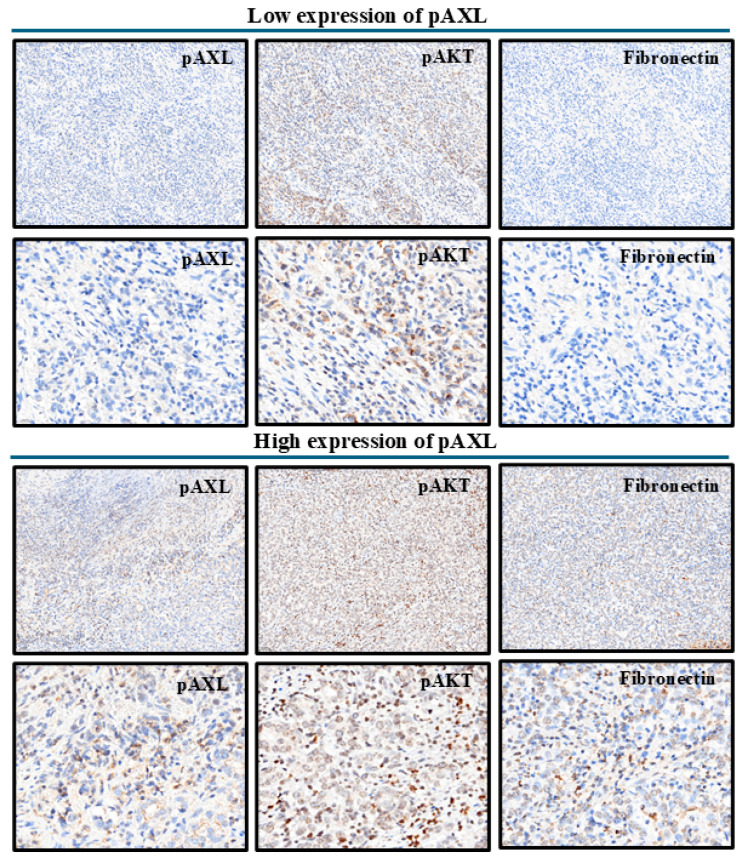
Schematic diagram of pAKT and fibronectin immunostaining in the low and high pAXL expression groups. The top two rows represent low pAXL expression, and the bottom two rows represent high pAXL expression. The scale bars are 500 µm and 200 µm, respectively.

In the univariate analysis, male patients had an odds ratio (OR) of 1.81 (95% confidence interval (CI): 0.96–3.41, *p* = 0.068), which became significant in the multivariate analysis with an OR of 2.26 (95% CI: 1.05–4.89, *p* = 0.038). Factors such as age, tumor grade, tumor size, and tumor stage were not significantly associated with the outcomes. Borderline associations were observed for CD31, N-cadherin, DNAJB4, MCRS1, and EhpA5DrTT in univariate analysis, but these were not significant. In contrast, fibronectin and pAkt expression levels were significantly associated with outcomes in both univariate (fibronectin OR: 1.02, 95% CI: 1.01–1.02, *p* < 0.001; pAkt OR: 1.04, 95% CI: 1.02–1.06, *p* < 0.001) and multivariate analyses (fibronectin OR: 1.02, 95% CI: 1.01–1.02, *p* < 0.001; pAkt OR: 1.04, 95% CI: 1.02–1.06, *p* < 0.001). Although pErk, pStat3, HMGA1, and IGF2BP1 were significant in the univariate analysis, they were not significant after adjustment in the multivariate analysis. Overall, fibronectin and pAkt were the only markers consistently and significantly associated with outcomes in both univariate and multivariate analyses, while other markers showed significance only in univariate analysis. When comparing the odds ratios of various markers between high and low pAXL expression, fibronectin and pAkt emerged as the markers most consistently associated with significant outcomes (Table 3 and Figure 3).

**Figure 4:**
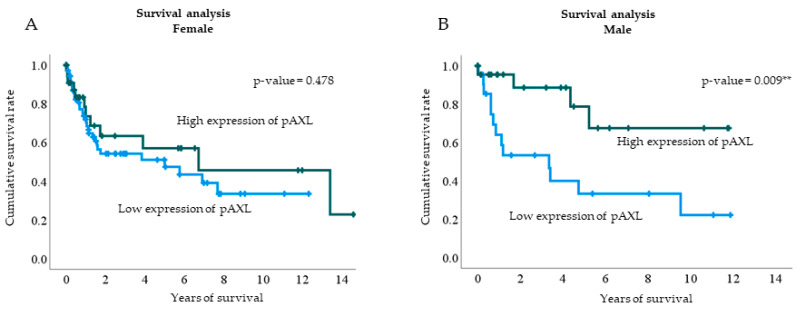
Kaplan–Meier survival analysis based on pAXL expression levels in female (**A**) and male patients (**B**). The blue line represents low pAXL expression, and the green line represents high pAXL expression. The *p*-values for female and male patients were 0.478 and 0.009, respectively. ** *p* < 0.01.

In the multivariate logistic regression analysis, pAXL expression was higher in male patients. Therefore, we divided the cohort into two groups based on sex to conduct a cumulative survival analysis based on pAXL expression levels. Male patients with higher pAXL expression levels had better survival rates (*p* = 0.009). Although a similar trend was observed in female patients, the difference was not significant (Figure 4).

## 4. Discussion

In the present study, we demonstrated that while several factors, including age, tumor grade, size, and stage, were not significantly associated with patient outcomes, fibronectin and pAkt expression levels emerged as the only markers consistently and significantly linked to outcomes in both univariate and multivariate analyses. Additionally, male sex was significantly associated with outcomes in the multivariate analysis. Other markers, such as pErk, pStat3, HMGA1, and IGF2BP1, showed significance in univariate analyses but lost this significance after adjustments. This finding highlights the robustness of fibronectin and pAkt as key prognostic indicators in GC.

Previous studies have underscored the critical role of the GAS6/AXL signaling axis in driving the aggressive behavior of gastric carcinoma cells, particularly through interactions with cancer-associated fibroblasts (CAFs). Inhibition of AXL, whether through genetic methods or pharmacological inhibitors like BGB324, can significantly diminish CAF-induced aggressiveness in GC cells, resulting in reduced cell motility, viability, and expression of markers linked to EMT [30]. However, our findings contrast with those of Bae et al., who reported that higher pAXL expression in human GC specimens was associated with poorer survival rates. Although their study explored the GAS6/AXL pathway through cell and animal experiments, it did not consider the background characteristics of the patients with GC. Our multivariate regression analysis indicated that male sex, fibronectin, and pAkt were independently correlated with higher pAXL expression, a result less frequently reported in previous studies. Below, we discuss the relationship between these three variables and pAXL expression.

Recent studies in male melanoma and male breast cancer (MBC) have identified significant roles played by the androgen receptor (AR) and AXL in cancer progression and prognosis [32]. In male melanoma, AR-positive patients exhibited worse survival rates compared to AR-negative patients, attributed to the ability of AR to enhance melanoma cell invasion through modulation of the MITF-AXL signaling pathway [33]. This modulation involves the alteration of miRNA-539-3p/USP13 signaling, leading to increased degradation of the MITF protein due to reduced deubiquitination. Consequently, AR promotes melanoma metastasis, a process that could potentially be counteracted by restoring MITF expression. This finding suggests that targeting AR-related signaling pathways with degradation enhancers, such as ASC-J9, could be a promising strategy for suppressing melanoma metastasis [33].

Similarly, in MBC, research has shown that the expression of the Hippo signaling transducer, TAZ/YAP, and its target gene, CTGF, correlates with poorer survival outcomes. AXL, identified as a transcriptional target of TAZ/YAP, was positively associated with TAZ/CTGF and YAP/CTGF phenotypes. Patients with tumors expressing these markers tend to have lower survival rates, and these phenotypes represent adverse factors for long-term survival, as demonstrated by multivariate Cox regression models. The expression pattern of AXL supports the notion that TAZ/YAP activation plays a detrimental role in MBC, further emphasizing the significance of Hippo-linked biomarkers in understanding and treating this rare disease [32].

Our results align with these findings, indicating that both MBC and ARs regulate AXL expression, leading to poorer prognosis. Based on multivariate logistic regression and Kaplan–Meier subgroup analyses, we found that pAXL expression was positively correlated with male sex. When stratified by sex, the analysis revealed that male patients exhibited higher pAXL expression and had better survival rates than female patients.

Fibronectin is one of the proteins involved in EMT; however, discussions about its relationship with AXL are limited. In this section, we focus on AXL and its interaction with EMT proteins. Antony et al. indicated that AXL inhibition reduces the expression of these transcription factors, thereby diminishing the invasiveness and metastatic potential of cancer cells [34]. In another study, AXL activation was found to induce EMT transcription factors such as SNAI1 (Snail), SNAI2 (Slug), and TWIST, which are crucial for downregulating epithelial markers (such as E-cadherin) and upregulating mesenchymal markers (such as vimentin) [35]. In our study, AXL activation upregulated EMT, consistent with the abovementioned results.

One of the key pathways through which AXL exerts its effects is the PI3K/AKT signaling pathway. Upon activation, AXL promotes the phosphorylation and activation of AKT, a critical kinase involved in cell survival and growth. AXL-driven AKT activation is associated with increased tumor aggressiveness and poor prognosis in various cancers, including hepatocellular carcinoma and non-small cell lung cancer. This activation contributes to oncogenic signaling, enhancing tumor cell survival and proliferation while making tumors more resistant to targeted therapies and conventional anticancer treatments. Our analysis indicates that increased pAKT expression is an independent factor contributing to elevated pAXL expression; however, further investigation is necessary to determine its correlation with survival rates [36,37,38].

### Limitations

This study has several limitations. Although trends were observed in the distribution of sex, age, and tumor grade, size, and stage between the low- and high-pAXL-expression groups, none of these differences were significant, potentially due to sample size or data variability, limiting the generalizability of the findings. The inconsistencies with the results of previous research, particularly regarding the prognostic value of pAXL expression, must also be highlighted. We acknowledge the need for further exploration of patient background characteristics and the relationship between fibronectin and AXL. Additionally, the correlation between pAXL expression and the male sex introduces a potential sex bias, which has not yet been fully explored. Finally, the need for further analysis to confirm the correlation between increased pAKT expression and survival rate suggests that these findings are preliminary and require further validation.

## 5. Conclusions

This study confirms phosphorylated AXL (pAXL) as a significant prognostic marker in gastric cancer (GC). Elevated pAXL expression was associated with improved survival, particularly among male patients, and correlated with increased levels of fibronectin and phosphorylated AKT, both involved in cancer invasiveness and epithelial–mesenchymal transition (EMT). These findings suggest that pAXL promotes GC progression through these molecular pathways and holds potential as a therapeutic target. Future research should further explore pAXL-targeted therapies, especially for male patients, to enhance treatment outcomes and better understand the role of sex-related differences in GC progression.

## Figures and Tables

**Table 1:** Comparison of patient characteristics and tumor features between low and high phosphorylated AXL (pAXL) expression groups.

	Low pAXL Expression	High pAXL Expression	Total	*p*-Value
Sex				0.067
Female	35 (28.0%)	26 (41.3%)	61 (32.4%)	
Male	90 (72.0%)	37 (58.7%)	127 (67.6%)	
Age	76 (63.8, 80.3)	72.5 (57.8, 80.0)	75.0 (63.0, 80.0)	0.144
Grading				0.835
Well	4 (3.3%)	3 (4.8%)	7 (3.8%)	
Moderate	30 (24.4%)	16 (25.8%)	46 (24.9%)	
Low	89 (72.4%)	43 (69.4%)	132 (71.4%)	
Size	4.5 (3.0, 6.5)	4.3 (2.0, 7.0)	4.5 (2.9, 7.0)	0.449
Stage				0.226
I and II	48 (38.4%)	30 (47.6%)	78 (41.5%)	
III and IV	77 (61.6%)	33 (52.4%)	110 (58.5%)	

**Table 2:** Comparison of biomarker expression levels between the low- and high-pAXL-expression groups. H-scores (Q1, Q3).

	Low pAXL Expression H-Scores (Q1, Q3)	High pAXL Expression H-Scores (Q1, Q3)	Total H-Scores (Q1, Q3)	*p*-Value
pAXL	1.55 (1.14, 2.16)	6.21 (4.26, 11.02)	2.16 (1.32, 4.27)	<0.001 ***
Caspase3	4.32 (3.13, 6.22)	4.65 (3.15, 10.11)	4.38 (3.15, 6.62)	0.662
Ki-67	13.16 (4.12, 35.98)	14.49 (2.39, 36.80)	13.61 (3.47, 36.00)	0.890
CD31	20.79 (12.15, 30.93)	22.68 (14.25, 38.05)	21.24 (13.08, 34.40)	0.135
E-cad	104.68 (100.97, 112.83)	106.92 (102.85, 112.56)	105.61 (101.83, 112.70)	0.113
N-cad	6.96 (3.23, 11.06)	6.88 (2.88, 16.00)	6.92 (3.04, 11.77)	0.778
Fibronectin	17.17 (1.10, 62.76)	94.66 (32.78, 141.36)	37.70 (3.61, 101.13)	<0.001 ***
pAkt	6.47 (2.93, 15.53)	17.21 (8.30, 40.91)	10.07 (3.79, 26.38)	<0.001 ***
pErk	0.71 (0.12, 2.37)	1.98 (0.49, 7.35)	0.92 (0.16, 3.33)	0.002 **
pStat3	0.09 (0.01, 0.65)	0.26 (0.05, 1.44)	0.16 (0.01, 0.75)	0.021 *
pAMPK	3.99 (2.94, 7.06)	3.37 (2.24, 5.53)	3.90 (2.64, 6.11)	0.030 *
DNAJB4	2.62 (1.56, 6.21)	2.64 (1.45, 7.14)	2.64 (1.56, 6.36)	0.626
HMGA1	4.19 (0.63, 23.43)	16.02 (3.52, 96.19)	6.65 (1.00, 44.95)	<0.001 ***
HOXA5	34.3 (11.74, 63.95)	33.66 (9.55, 66.72)	6.65 (1.01, 44.95)	0.901
IGF2BP1	45.81 (22.59, 66.37)	35.66 (9.55, 66.72)	42.97 (19.11, 63.56)	0.014 *
MCRS1	108.35 (72.15, 160.10)	93.52 (67.89, 130.57)	103.45 (70.49, 146.88)	0.105
EhpA5DrTT	6.96 (3.29, 19.67)	9.48 (3.12, 21.50)	7.65 (3.20, 19.82)	0.389

* *p* < 0.05, ** *p* < 0.01, *** *p* < 0.001.

**Table 3:** Univariate and multivariate analyses.

	Univariate	*p*-Value	Multivariate	*p*-Value
Sex				
Female	Reference		Reference	
Male	1.81 (0.96–3.41)	0.068	2.26 (1.05–4.89)	0.038 *
Age	0.98 (0.96–1.01)	0.118	1.00 (0.97–1.03)	0.891
Grading				
Well	Reference			
Moderate	0.71 (0.14–3.58)	0.679		
Low	0.64 (0.14–3.01)	0.576		
Size	1.01 (0.91–1.12)	0.900		
Stage				
I and II	Reference			
III and IV	0.69 (0.37–1.26)	0.227		
Marker				
caspase3	1.02 (0.98–1.07)	0.291		
Ki67	1.01(1.00–1.02)	0.275		
CD31	1.02 (1.00–1.03)	0.056		
E-cad	1.01 (0.98–1.05)	0.382		
N-cad	1.02 (1.00–1.04)	0.082		
Fibronectin	1.02 (1.01–1.02)	<0.001 **	1.02 (1.01–1.02)	<0.001 ***
pAkt	1.04 (1.02–1.06)	<0.001 ***	1.04 (1.02–1.06)	<0.001 ***
pErk	1.02 (1.00–1.03)	0.033 *	1.01 (0.98–1.03)	0.624
pStat3	1.06 (1.01–1.12)	0.020 *	1.03 (0.96–1.10)	0.388
pAMPK	0.99 (0.95–1.04)	0.765		
DNAJB4	1.03 (1.00–1.07)	0.092		
HMGA1	1.01 (1.00–1.01)	0.006 **	1.00 (0.99–1.01)	0.686
HOXA5	1.00 (0.99–1.01)	0.902		
IGF2BP1	0.99(0.98–1.00)	0.030 *	1.00 (0.98–1.01)	0.636
MCRS1	0.99 (0.99–1.00)	0.078		
EhpA5DrTT	1.01 (1.00–1.03)	0.071		

* *p* < 0.05, ** *p* < 0.01, *** *p* < 0.001.

## Data Availability

Data can be obtained from the corresponding author upon request.

## Supplementary Materials

The following supporting information can be downloaded at: https://www.mdpi.com/article/10.3390/jcm13226694/s1, Figure S1: The immunohistochemistry images of various biomarkers with high and low pAXL expression levels.; Table S1: Details of primary antibodies used in this study.
