# Supplementary material for: Association Between Phosphorylated AXL Expression and Survival in Patients with Gastric Cancer
Source: J Clin Med. 2024 Nov 7;13(22):6694. doi: 10.3390/jcm13226694 (PMC11595014; doi:10.3390/jcm13226694)
Supplement: Supplementary file 1 [file jcm-13-06694-s001.zip › Supplementary_Table_1.pdf]

**Supplementary Table S1.** Details of primary antibodies used in ths study.

| | Brand | Catalog number | Dilution ratio |
|---|---|---|---|
| pAXL | R&D | AF2228 | 1:100 |
| Caspase3 | Cell Signaling | 9664 | 1:100 |
| Ki67 | BioLegend | 350503 | 1:100 |
| CD31 | Abbiotec | 250590 | 1:500 |
| E-cad | Abcam | ab40772 | 1:100 |
| N-cad | Abcam | ab76011 | 1:100 |
| Fibronectin | Santa Cruz | SC-8422 | 1:50 |
| pAkt | GeneTex | GTX11901 | 1:50 |
| pErk | R&D | AF1018 | 1:200 |
| pStat3 | Abcam | ab76315 | 1:50 |
| pAMPK | Cell signal | 2535 | 1:100 |
| DNAJB4 | Novus | NBP1-81735 | 1:25 |
| HMGA1 | cell signal | #12094 | 1:50 |
| HOXA5 | Abcam | ab82645 | 1:50 |
| IGF2BP1 | Abcam | ab82968 | 1:75 |
| MCRS1 | Sigma | HPA039057 | 1:400 |
| EhpA5DrTT | Thermo | PA5-14581 | 1:200 |
