# Supplementary figures and images for: Association Between Phosphorylated AXL Expression and Survival in Patients with Gastric Cancer
Source: J Clin Med. 2024 Nov 7;13(22):6694. doi: 10.3390/jcm13226694 (PMC11595014; doi:10.3390/jcm13226694)

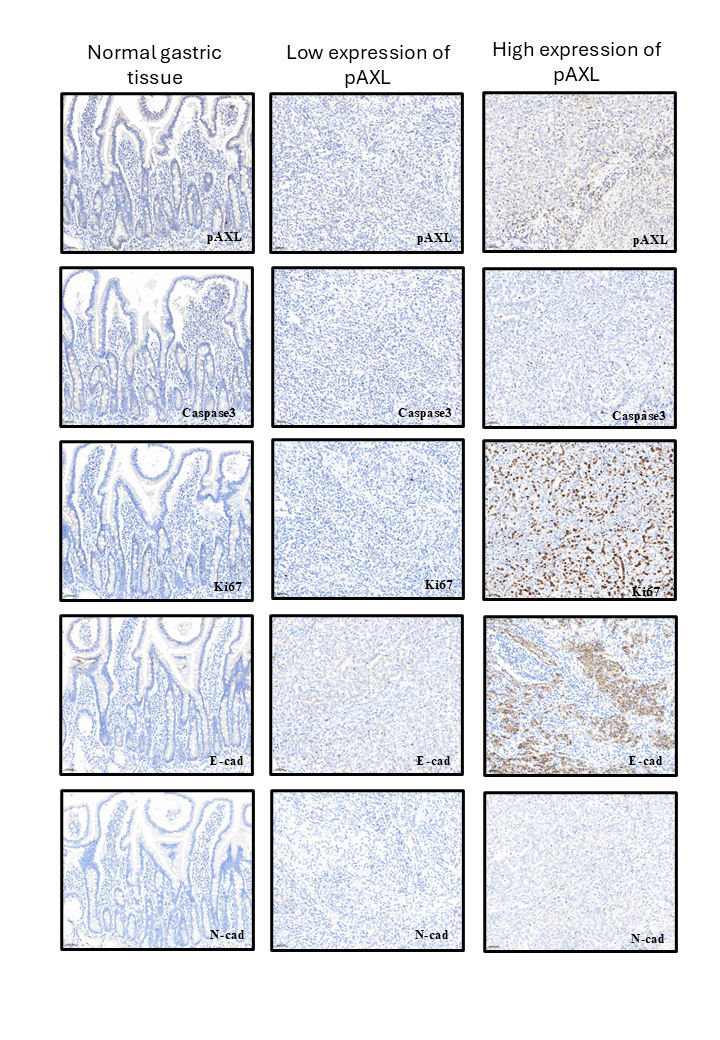

Supplement: Supplementary file 1 [file jcm-13-06694-s001.zip › Supplementary_Figure_1/figure S1a.TIF]

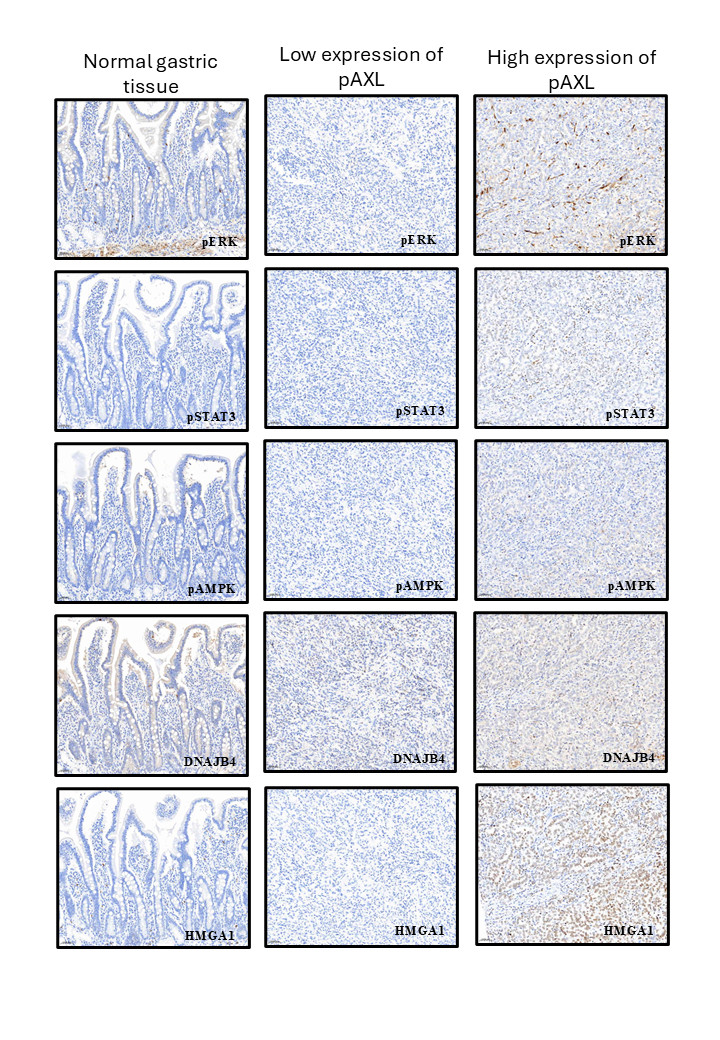

Supplement: Supplementary file 1 [file jcm-13-06694-s001.zip › Supplementary_Figure_1/figure S1b.TIF]

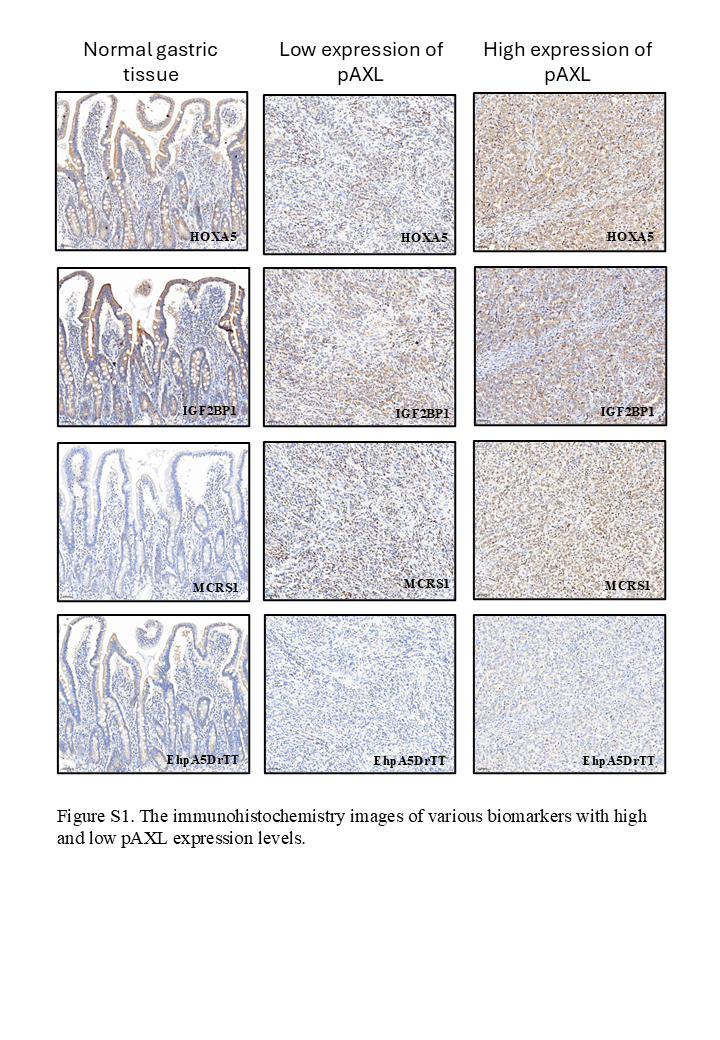

Supplement: Supplementary file 1 [file jcm-13-06694-s001.zip › Supplementary_Figure_1/figure S1c.TIF]
